# Adenosine Monophosphate Improves Lipolysis in Obese Mice by Reducing DNA Methylation via ADORA2A Activation by Ecto‐5′‐Nucleotidase (CD73)

**DOI:** 10.1002/advs.202405079

**Published:** 2025-02-20

**Authors:** Zhijuan Cui, Li Feng, Sujuan Rao, Zihao Huang, Shuangbo Huang, Liudan Liu, Yuan Liao, Zheng Lan, Qiling Chen, Jinping Deng, Leli Wang, Yulong Yin, Chengquan Tan

**Affiliations:** ^1^ State Key Laboratory of Swine and Poultry Breeding Industry Guangdong Provincial Key Laboratory of Animal Nutrition Control National Engineering Research Center for Breeding Swine Industry College of Animal Science South China Agricultural University Guangzhou 510642 China; ^2^ Key Laboratory of Agro‐ecological Processes in Subtropical Region Institute of Subtropical Agriculture Chinese Academy of Sciences, Hunan Provincial Key Laboratory of Animal Nutritional Physiology and Metabolic Process Hunan Provincial Engineering Research Center for Healthy Livestock and Poultry Production Changsha 410125 China

**Keywords:** adenosine monophosphate, ADORA2A, DNA methylation, ecto‐5′‐nucleotidase, obesity

## Abstract

The previous work discovers the potential of adenosine monophosphate (AMP) to alleviate obesity‐related metabolic diseases, but the underlying molecular mechanisms remain incompletely understood. Here, AMP is confirmed to enhance white fat decomposition and improve abnormal glucose and lipid metabolism in mice fed with a high‐fat (HF) diet. Mechanically, AMP is converted to adenosine (ADO) through ecto‐5′‐nucleotidase (CD73), and adenosine A2A receptor (ADORA2A) signaling activation is involved in the down‐regulation of methylation in white adipose tissue, thereby reducing the hormone‐sensitive lipase (HSL) methylation level and promoting HSL transcription and white fat decomposition. Moreover, the metabolic benefits of AMP are found to be partially eliminated in ADORA2A knockout mice, but re‐expression of ADORA2A can reproduce the AMP‐induced metabolic regulation in white fat. These findings reveal the mechanism that AMP, as the upstream of ADO, stimulates ADORA2A signaling and white fat DNA methylation to participate in the anti‐obesity effect.

## Graphical abstract

In this study, the improvement effect of AMP on obesity can be attributed to the conversion of extracellular AMP to ADO through ADORA2A activation by CD73, which can promote lipolysis by reducing DNA methylation level and enhancing HSL gene transcription. These findings show the great potential of AMP in improving obesity.

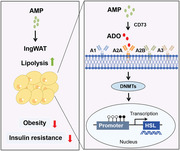

## 1 Introduction

With the rapid advancement of globalization and the economy, the ongoing rise in global obesity rates has become an increasingly concerning public health issue.^[^ ^1^, ^2^ ^]^ Obesity is a chronic and progressive condition that disrupts the energy balance of the entire body, leading to local and systemic metabolic disorders,^[^ ^3^ ^]^ and increasing the risk of various diseases, such as hypertension, hyperlipidemia, and type 2 diabetes.^[^ ^4^ ^]^ The main characteristic of obesity is excessive fat accumulation in organs,^[^ ^4^ ^]^ especially in white adipose tissue.^[^ ^5^ ^]^ There is growing evidence that promoting white fat browning can enhance fat oxidation, elevate energy expenditure, and ameliorate obesity.^[^ ^6^, ^7^ ^]^

Adenosine monophosphate (AMP), a compound composed of one molecule of adenosine and one phosphate, can be generated by adenosine triphosphate (ATP) hydrolysis and adenosine (ADO) metabolism.^[^ ^8^, ^9^, ^10^ ^]^ In our previous study, the supplementation of AMP with basal diet could improve abnormal glucose metabolism and reduce fat weight in mice,^[^ ^11^ ^]^ but the related mechanism remains unclear. Studies have shown that AMP can enhance the activation of AMP‐activated protein kinase (AMPK), which plays a crucial role in maintaining energy homeostasis and lipid metabolism.^[^ ^12^, ^13^ ^]^ Additionally, extracellular AMP can be converted into ADO by extracellular 5′‐ nucleotidase (CD73),^[^ ^14^ ^]^ and the enhancement of CD73 activity contributes to the increase of extracellular ADO level. ADO is an important cellular signal molecule that regulates various physiological processes by binding to its receptors, including glucose homeostasis, inflammation, adipogenesis, and insulin resistance.^[^ ^15^ ^]^ Moreover, ADO was reported to have the potential to decrease DNA methylation,^[^ ^16^ ^]^ which plays a significant role in obesity occurrence and development.^[^ ^17^, ^18^ ^]^ The variations in DNA methylation levels between obese individuals and those with normal weight may exert an impact on adipocyte function.^[^ ^19^, ^20^, ^21^ ^]^ These reports suggest the necessity to explore whether AMP and its conversion product ADO can regulate fat methylation levels for developing strategies to alleviate obesity.

Therefore, the current study aims to investigate the potential impact of AMP, a precursor of ADO, on DNA methylation levels and lipid metabolism in adipocytes. Our findings demonstrate that AMP can effectively reduce body weight and improve glucose and lipid metabolism in mice fed with a high‐fat (HF) diet. Additionally, the anti‐obesity effects of AMP were found to be mediated through its conversion into ADO by CD73. Mechanistically, AMP promotes fat decomposition by reducing DNA methylation via adenosine receptor (ADORA2A) signaling, leading to an increase in hormone‐sensitive lipase (HSL) level. This study sheds light on the effects of AMP on DNA methylation and lipid metabolism to provide novel insights for combating obesity.

## 2 Results

### 2.1 Exogenous AMP Supplementation Improves the Metabolic Phenotype of Obese Mice

**Figure 1:**
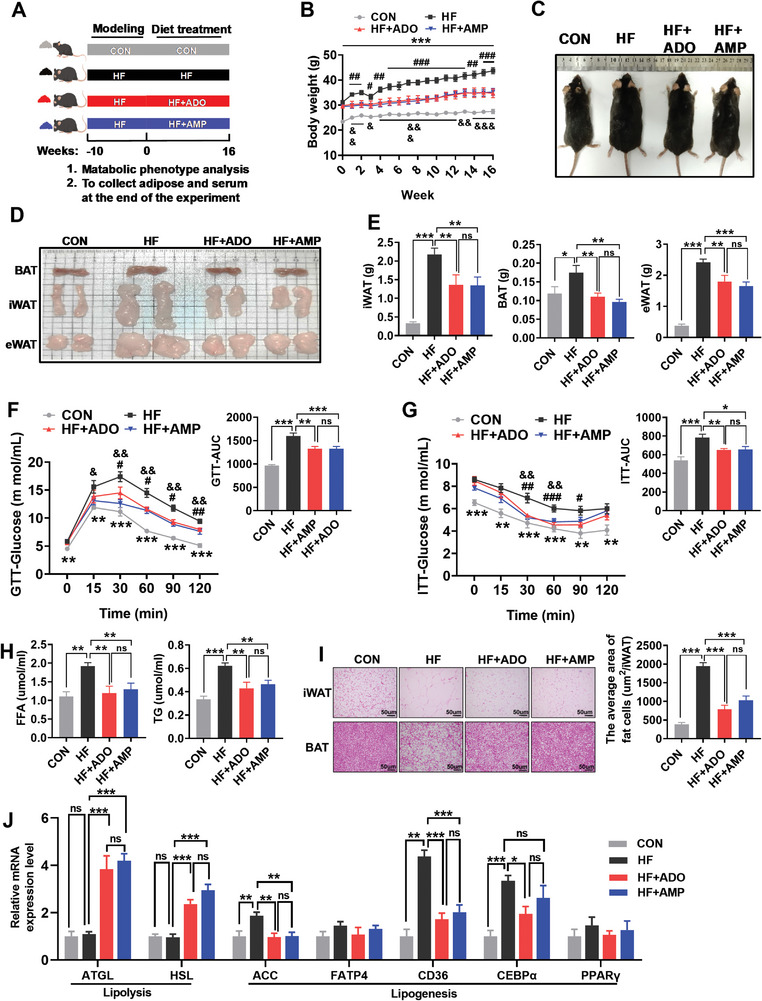
Exogenous AMP supplementation improves the metabolic phenotype of obese mice A) The schematic diagram of induction and treatment of obese mice. Before the formal trial, C57BL/6 mice were fed separately with a control diet (*n* = 11) and a high‐fat diet (*n* = 33) for 10 weeks. Then, the control group was further fed with control diet (CON) (*n* = 11), and the obese mice were fed separately with high‐fat diet (HF) (*n* = 11), high‐fat diet + 0.1% ADO (HF+ADO) (*n* = 11), and high‐fat diet + 0.1% AMP (HF+AMP) (*n* = 11) for 16 weeks. B) The changes in body weight of mice within 0–16 weeks after the formal test (*n* = 11). C) The schematic diagram of mice before slaughter. D) The schematic diagrams of BAT, iWAT, and eWAT in the four groups. E) The weight of BAT, iWAT, and eWAT in the four groups (*n* = 8). F) GTT and AUC at the end of the experiment (*n* = 6). After fasting overnight and then intraperitoneal injection with glucose 2 g kg^−1^, the blood glucose of the mice was measured and AUC was calculated at the specified time point (*n* = 6). G) ITT and AUC at the end of the experiment (*n* = 6). After fasting for 5 h and then injection with insulin 0.75 IU kg^−1^, the blood glucose of the mice was measured and AUC was calculated at the specified time point (*n* = 6). H) The content of FFA and TG in the serum of the four groups (*n* = 6). I) The HE staining of iWAT and BAT and the fat area in iWAT (*n* = 6). J) Real‐time fluorescence quantification of lipid metabolism genes in iWAT (*n* = 6). Data were analyzed by one‐way ANOVA followed by post hoc Tukey's tests and represented mean ± SEM. In Figure 1B,F–G, ^*^, ^#^, and ^&^ represent the comparison of HF with CON, HF+ADO and HF+AMP, respectively. ^*^ *p <* 0.05, ^**^ *p <* 0.01, ^***^ *p <* 0.001, ^#^ *p <* 0.05, ^##^ *p <* 0.01, ^&^ *p <* 0.05, and ^&&^ *p <* 0.01. ACC, acetyl‐CoA carboxylase; ADO, adenosine; AMP, adenosine monophosphate; ATGL, adipose triglyceride lipase; AUC, area under curve; BAT, brown fat; CD36, CD36 molecule; CEBPα, CCAAT‐enhancer‐binding protein alpha; eWAT, epididymal white adipose tissue; FATP4, fatty acid transporter protein 4; FFA, free fatty acid; GTT, glucose tolerance test; HSL, hormone‐sensitive lipase; ITT, insulin tolerance test; iWAT, inguinal white adipose tissue; PPARγ, peroxisome proliferator‐activated receptorγ; TG, triglyceride.

The construction of high‐fat diet‐induced obese (DIO) mice and the dietary treatments are shown in **Figure** **1**A. Compared to the HF group, the groups treated with ADO or AMP showed similar food intake (Figure S1A, Supporting Information). Interestingly, consistent with the results of ADO supplementation, AMP supplementation reduced weight gain and adiposity in the HF group (Figure 1B,C). Cold exposure revealed that both ADO and AMP supplementation could increase core temperature in the rectum and brown fat (BAT) in the HF group (Figure S1B–D, Supporting Information). Additionally, ADO could significantly elevate the minimum, maximum, and average energy consumption in HF group, whereas AMP could increase the average energy consumption throughout the day (Figure S1E–G, Supporting Information). Notably, both ADO and AMP reduced the weight of BAT, inguinal white adipose tissue (iWAT), and epididymal white adipose tissue (eWAT) in the HF group (Figure 1E), but had no effect on muscle weights (Figure S1H–K, Supporting Information). To determine the effects of AMP treatment on glucose metabolism, glucose tolerance test (GTT) and insulin tolerance test (ITT) were conducted in all experimental groups. Compared to CON‐fed mice, the HF‐fed mice showed higher glucose tolerance and insulin resistance (Figure 1F,G). However, ADO and AMP supplementation could significantly improve glucose tolerance (Figure 1F) and insulin resistance in the HF group (Figure 1G). Moreover, HF‐fed mice also suffered from dyslipidemia, with a significant increase in serum levels of free fatty acids (FFA) and triglycerides (TG), while AMP and ADO addition could also reduce the serum levels of FFA and TG (Figure 1H). Histopathological analysis of iWAT and BAT found that both ADO and AMP could reduce the increase of fat area caused by obesity (Figure 1I). Then we examined the expression of genes related to lipid decomposition and synthesis in iWAT, and we found that ADO and AMP could increase the levels of adipose triglyceride lipase (*ATGL*) and *HSL* mRNA while decreasing the levels of acetyl‐CoA carboxylase (*ACC*) and CD36 molecule (*CD36*) mRNA (Figure 1J). These data suggest that AMP can improve the metabolic phenotype of HF‐fed mice in the same way as ADO.

### 2.2 CD73 Promotes Transformation of AMP into ADO

**Figure 2:**
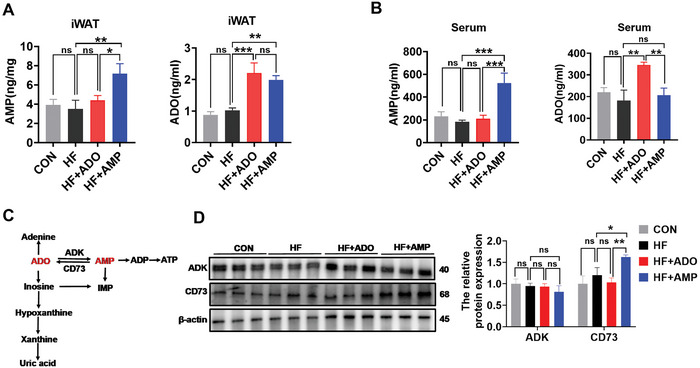
CD73 promotes the transformation of AMP into ADO A) The content of AMP and ADO in iWAT (*n* = 6). B) The content of AMP and ADO in serum (*n* = 6). C) The schematic diagram of metabolic conversion between AMP and ADO. D) Western blotting and quantification of CD73 and ADK in iWAT (*n* = 6). Data were analyzed by one‐way ANOVA followed by post hoc Tukey's tests and presented as mean ± SEM. ^ns^ *p* > 0.05, ^*^ *p <* 0.05, and ^**^ *p <* 0.01. ADK, adenosine kinase; ADO, adenosine; ADP, adenosine diphosphate; AMP, adenosine monophosphate; ATP, adenosine triphosphate; CD73, ecto‐5′‐nucleotidase; iWAT, inguinal white adipose tissue; IMP, inosine 5′‐monophosphate.

The metabolic effects of ADO and AMP were investigated by using liquid chromatography‐mass spectrometry to measure the levels of ADO and AMP in iWAT and serum of HF‐fed mice. In **Figure** **2**, we found no significant differences in the levels of ADO and AMP in iWAT and serum between CON group and the HF group, as well as no differences of adenosine kinase (ADK) and CD73 protein in iWAT (Figure 2A–D). As expected, compared with HF group, ADO or AMP supplementation could significantly elevate the level of ADO in iWAT, while ADO supplementation did not alter the level of AMP (Figure 2A). Consistent with the results in tissue, AMP supplementation could also slightly but not significantly increase the level of ADO in serum, while ADO supplementation still failed to cause any change in the level of AMP in serum (Figure 2B). Following AMP supplementation, xanthine showed a significant increase in iWAT, in contrast to no significant difference in serum among the downstream metabolites of xanthine, adenine, hypoxanthine, and uric acid (Figure S2A–H, Supporting Information). These findings prompt further investigation into whether the conversion of AMP to ADO plays a beneficial role in anti‐obesity. Given that ADK can convert ADO to AMP,^[^ ^8^, ^9^ ^]^ while CD73 can convert AMP to ADO (Figure 2C),^[^ ^22^ ^]^ we subsequently assessed the protein levels of ADK and CD73 in iWAT. Further western blotting analysis showed an increase in the CD73 protein level in HF‐AMP mice, which promoted the conversion of AMP to ADO (Figure 2D). Therefore, we speculate that AMP supplementation may increase ADO levels only through CD73.

### 2.3 AMP is Metabolized into ADO by CD73 to Play an Anti‐Obesity Role

**Figure 3:**
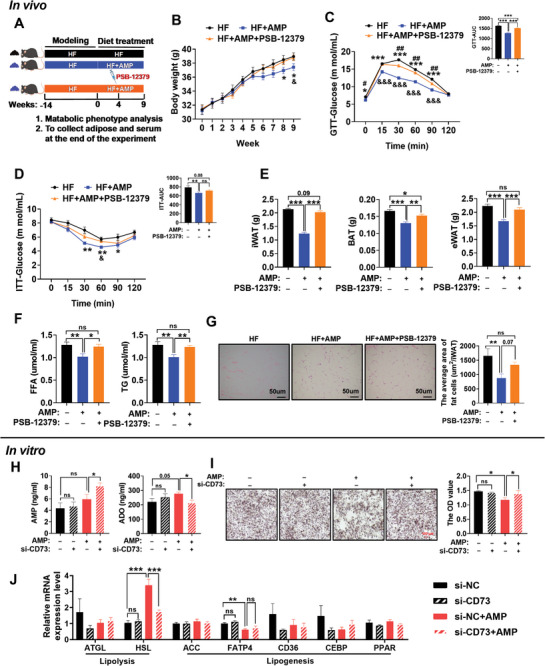
AMP is metabolized into ADO by CD73 to play an anti‐obesity role. A) Experimental diagram of injection of CD73 inhibitor (30 mg kg^−1^ PSB‐12379) after AMP supplementation in DIO mice. B) The changes of body weight in 0–9 weeks (*n* = 6–8).C) GTT and AUC at the end of the experiment. After fasting overnight and intraperitoneal injection with glucose 2 g kg^−1^, the blood glucose of the mice was measured and AUC was calculated at the specified time point (*n* = 6–8). D) ITT and AUC at the end of the experiment (*n* = 5–6). After fasting for 5 h and then injection with insulin 0.75 IU kg^−1^, the blood glucose of the mice was measured, and AUC was calculated at the specified time point (*n* = 5–6). E)The weight of BAT, iWAT, and eWAT in each group (*n* = 6–7). F) The FFA and TG content in serum (*n* = 6–8). G) HE staining and fat area of iWAT (*n* = 6). H) The contents of AMP and ADO in 3T3‐L1 cells after CD73 knockdown (*n* = 5–6), respectively. I) Lipid droplet production and statistics of 3T3‐L1 cells after CD73 knockdown (*n* = 3). J) Real‐time fluorescence quantification of lipid metabolism genes in 3T3‐L1 cells after CD73 knockdown (*n* = 6). Data were analyzed by one‐way ANOVA followed by post hoc Tukey's tests and presented as mean ± SEM. In Figure 3B–D, ^*^, ^&^ and ^#^ represent the comparison of HF versus AMP, HF+AMP versus HF+AMP+PSB‐12379, and HF versus HF+AMP+PSB‐12379 respectively. ^ns^ *p* > 0.05, ^*^ *p <* 0.05, ^**^ *p <* 0.01, ^***^ *p <* 0.001, ^#^ *p <* 0.05, ^##^ *p <* 0.01, ^&^ *p <* 0.05, and ^&&^ *p <* 0.01. ACC, acetyl‐CoA carboxylase; ADO, adenosine; AMP, adenosine monophosphate; ATGL, adipose triglyceride lipase; AUC, area under the curve; BAT, brown fat; CD36, CD36 molecule; CD73, ecto‐5′‐nucleotidase; CEBPα, CCAAT‐enhancer‐binding protein alpha; eWAT, epididymal white adipose tissue; FATP4, fatty acid transporter protein 4; FFA, free fatty acid; GTT, glucose tolerance test; HSL, hormone‐sensitive lipase; ITT, insulin tolerance test; iWAT, inguinal white adipose tissue; PPARγ, peroxisome proliferator‐activated receptor γ; TG, triglyceride.

The role of endogenous CD73 as a key mediator in improving obesity metabolism under AMP treatment was verified by analyzing the metabolic phenotype of HF‐fed mice treated with CD73 inhibitor (PSB‐12379). The treatments of AMP and CD73 inhibitor are shown in **Figure** **3**A, and CD73 inhibitor could effectively reduce the CD73 protein level in iWAT (Figure S3A, Supporting Information). Consistent with the above results, AMP supplementation could significantly reduce the weight of HF‐fed mice, but this effect was eliminated after injection of CD73 inhibitor, resulting in no difference in their body weight versus HF mice (Figure 3B). Meanwhile, except that the group injected with the CD73 inhibitor was lower than the HF group at weeks 2 and 6, the three groups of mice showed no differences in feed intake (Figure S3B, Supporting Information). Further analysis revealed that the beneficial effects of AMP on glucose intolerance were attenuated by the CD73 inhibitor treatment, although the effect on insulin resistance was slight (Figure 3C,D). Similarly, the injection of CD73 inhibitor after AMP supplementation led to abnormal blood lipid metabolism in mice, as evidenced by increased serum levels of FFA and TG similar to the observations in the HF group (Figure 3F). As expected, AMP supplementation could reduce fat accumulation in BAT, iWAT, and eWAT, but this effect was reversed by CD73 inhibitor injection (Figure 3E). However, AMP can reduce the weight of SOL while neither AMP supplementation nor CD73 inhibitor injection showed any impact on muscle tissue (Figure S3C–F, Supporting Information). Histopathological analysis indicated that the average adipocyte size was smaller in iWAT treated with AMP, while CD73 inhibitors could eliminate the above result (Figure 3G).

The metabolic benefits of AMP were further confirmed by CD73 knockdown and culture of 3T3‐L1 cells with CD73 inhibitor (PSB‐12379), and these effects were found to vary with CD73 expression. Our results suggest that AMP supplementation could increase the protein level of CD73, but this effect was blocked by CD73 knockdown and CD73 inhibitor (Figure S3G,J, Supporting Information). Consistently, AMP supplementation increased the level of ADO in 3T3‐L1 cells, which was eliminated by CD73 inhibitor and CD73 knockdown (Figure 3H; Figure S3H, Supporting Information), while ADO supplementation had no effect on the level of AMP in 3T3‐L1 cells (Figure S3H, Supporting Information). Further oil red O staining showed that AMP and ADO could inhibit the production of lipid droplets, which however was increased by CD73 knockdown and CD73 inhibitor. After extracting the oil red O with isopropanol, quantitative analysis revealed a significant decrease in the optical density (OD) value of AMP and ADO versus the control, while a significant increase in the OD value of si‐CD73 plasmid and CD73 inhibitor versus AMP (Figure 3I, Figure S3I, Supporting Information). Additionally, consistent with our findings from in vivo studies, AMP supplementation increased the level of HSL related to lipolysis, while this effect was eliminated by CD73 knockdown (Figure 3J). All these results collectively confirmed the involvement of CD73 in the regulation of lipid metabolism by AMP.

### 2.4 AMP Elevates HSL levels by Reducing DNA Methylation through CD73 Expression

**Figure 4:**
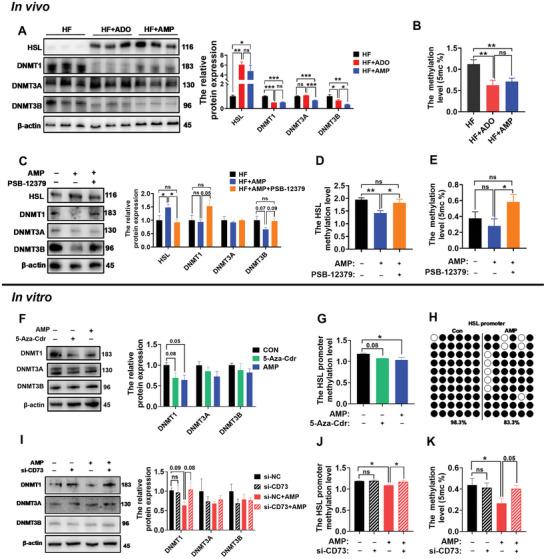
AMP elevates HSL levels by reducing DNA methylation through CD73 expression. A) Western blotting and quantification of DNMTs and HSL in iWAT (*n* = 3). B) Global DNA methylation level in iWAT (*n* = 6). C) Western blotting and quantification of DNMTs and HSL in iWAT after injection of CD73 inhibitor (30 mg kg^−1^ PSB‐12379) (*n* = 3). D) HSL promoter methylation level in iWAT after injection of CD73 inhibitor (30 mg kg^−1^ PSB‐12379) (*n* = 6). E) Global DNA methylation level in iWAT after injection of CD73 inhibitor (30 mg kg^−1^ PSB‐12379) (*n* = 6). F) Western blotting and quantification of DNMTs in 3T3‐L1 cells after adding 10 µm 5‐Aza‐Cdr and 1 µm AMP (*n* = 3), respectively. G) HSL promoter methylation level in 3T3‐L1 cells after adding 10 µm 5‐Aza‐Cdr and 1 µm AMP (*n* = 3), respectively. H) Bisulfite Sequencing PCR analysis of ten clones for each group of samples. I) Western blotting and quantification of DNMTs in 3T3‐L1 cells after CD73 knockdown (*n* = 3). J) HSL methylation level in 3T3‐L1 cells after CD73 knockdown (*n* = 3). K) Global DNA methylation level in 3T3‐L1 cells after CD73 knockdown (*n* = 6). Data were presented as mean ± SEM and analyzed by one‐way ANOVA, followed by post hoc Tukey's tests (Figure 4 A–G, I) or unpaired Student's t‐test (Figure 4H). ^ns^ *p* > 0.05, ^*^ *p <* 0.05, and ^**^ *p <* 0.01. 5mc, 5‐methylcytosine; AMP, adenosine monophosphate; ADO, adenosine; CD73, ecto‐5′‐nucleotidase; DNMT1, DNA methyltransferase 1; DNMT3A, DNA methyltransferase 3A; DNMT3B, DNA methyltransferase 3B HSL, hormone‐sensitive lipase; iWAT, inguinal white adipose tissue.

ADO has been reported to reduce DNA methylation, which plays a significant role in obesity occurrence and development.^[^ ^17^, ^18^ ^]^ In order to explore the potential mechanism of AMP treatment for controlling weight loss in HF‐fed mice, we tested DNA methyltransferases (DNMTs) in mouse iWAT. Interestingly, ADO and AMP could decrease the protein levels of DNMT1 and DNMT3B, while increasing the protein level of HSL in iWAT (**Figure** **4**A,C). Both ADO and AMP reduced the global DNA methylation level in iWAT (Figure 4B), but showed no significant difference in BAT (Figure S4A, Supporting Information). Since DNA hypomethylation is related to gene activation, we detected the methylation level of HSL gene promoters according to the increased mRNA expression of *HSL* in vivo (Figure 1J) and in vitro (Figure 3J). We found that blocking CD73 could eliminate the reducing effect of AMP on HSL promoter methylation and global DNA methylation level (Figure 4D,E). This prompted us to verify the relationship between AMP and DNA methylation in 3T3‐L1 cells using a DNA methylase inhibitor (5‐Aza‐Cdr). We found that both 5‐Aza‐Cdr could increase HSL expression and reduce the global DNA methylation level while AMP could inhibit lipid droplet formation and increase HSL expression level (Figure S4B–D, Supporting Information). Consistent with the therapeutic effect of 5‐Aza‐Cdr, AMP could decrease DNMT1 protein and HSL promoter methylation levels in 3T3‐L1 cells (Figure 4F,G, Supporting Information). Additionally, we analyzed the methylation site of the HSL promoter by Bisulfite Sequencing PCR (BSP) and found that AMP supplementation could reduce the HSL promoter methylation level (Figure 4H). Moreover, we explored the direct connection between CD73 and DNA methylation on 3T3 cells by CD73 knockdown and using CD73 inhibitors. We found that the reducing effect of AMP on DNMT1 was reversed under conditions of reduced CD73 expression (Figure 4I). Furthermore, AMP could no longer reduce the level of HSL promoter methylation or global DNA methylation level after reducing CD73 expression in 3T3‐L1 cells (Figure 4J,K). Meanwhile, we also observed that the above effects of AMP could be eliminated by CD73 inhibitor (Figure S4E–G, Supporting Information). All these observations indicate that AMP elevates HSL level by reducing DNA methylation in iWAT through CD73 expression, thereby promoting lipolysis.

### 2.5 ADORA2A is Involved in HSL Promoter Methylation Reduction by AMP

**Figure 5:**
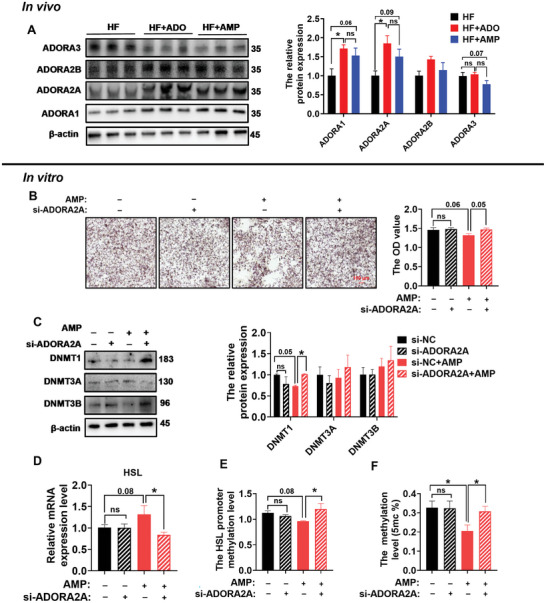
ADORA2A is involved in HSL promoter methylation reduction by AMP. A) Western blotting and quantification of ADORA1, ADORA2A, ADORA2B, and ADORA3 in iWAT (*n* = 3). B) Lipid droplet formation and statistics of 3T3‐L1 cells after ADORA2A knockdown (*n* = 3). C) Western blotting and quantification of DNMTs in 3T3‐L1 cells after ADORA2A knockdown (*n* = 3). D) Real‐time fluorescence quantification of HSL in 3T3‐L1 cells after ADORA2A knockdown (*n* = 6). E) HSL promoter methylation level in 3T3‐L1 cells after ADORA2A knockdown (*n* = 3). F) Global DNA methylation level in 3T3‐L1 cells after ADORA2A knockdown (*n* = 6). Data were analyzed by one‐way ANOVA followed by post hoc Tukey's tests and presented as mean ± SEM. ^ns^ *p* > 0.05, ^*^ *p <* 0.05, and ^**^ *p <* 0.01. 5mc, 5‐methylcytosine; ADO, adenosine; AMP, adenosine monophosphate; DNMT1, DNA methyltransferase 1; DNMT3A, DNA methyltransferase 3A; DNMT3B, DNA methyltransferase 3B; HSL, hormone‐sensitive lipase; iWAT, inguinal white adipose tissue.

AMP can be converted to ADO through CD73, and ADO can be transported into cells by nucleoside transporter or play a role through activation of downstream pathways by adenosine receptors. However, we found that nucleoside transporter knockdown did not eliminate the inhibitory effect of AMP on lipid droplet formation (Figure S5A, Supporting Information). ADO has been reported to participate in the anti‐obesity through four adenosine receptors,^[^ ^15^ ^]^ so we further investigated the impact of AMP on ADO receptors. Compared with HF group, AMP increased the ADORA2A and ADORA1 protein levels and decreased the ADORA3 protein level (**Figure** **5**A). Since ADORA1 has been reported to be involved in lipogenesis,^[^ ^15^, ^23^, ^24^ ^]^ we paid more attention to ADORA2A and ADORA3. We explored the role of different adenosine receptors in the anti‐obesity effect of AMP by ADORA2A knockdown and ADORA3 overexpression through drug therapy. The involvement of ADORA2A and ADORA3 in the lipid metabolism process under the influence of AMP was investigated by treating 3T3‐L1 cells with an ADORA2A inhibitor (10 µm M241385) and an ADORA3 agonist (10 µm Namodenoson). First, both ADORA2A inhibitor and ADORA3 agonist were shown to effectively inhibit and increase the protein levels of ADORA2A and ADORA3, respectively (Figure S5C, Supporting Information). In oil red O staining, both ADORA2A inhibitor and ADORA3 agonist were observed to counteract the inhibitory effect of ADO on lipid droplet formation, leading to an increase in OD value after isopropanol extraction (Figure S5B, Supporting Information). Although activation of ADORA3 reversed ADO decrease in HSL and DNMTs expression, only after blocking ADORA2A, could the effect of ADO on HSL promoter methylation, global methylation, and DNMTs be reversed (Figure S5C–E, Supporting Information). Consistently, after ADORA2A knockdown in 3T3‐L1 cells, the inhibitory effect of AMP on lipid droplet formation was eliminated (Figure 5B). Additionally, AMP could reduce DNMT1 protein levels and conversely increase HSL gene level, but these effects were reversed by ADORA2A knockdown (Figure 5C,D). Moreover, the reducing effect of AMP on HSL promoter methylation level and global DNA methylation level was also eliminated after ADORA2A knockdown (Figure 5E,F). These findings indicate that the ADORA2A pathway plays a crucial role in mediating AMP‐induced changes in the DNA methylation level.

### 2.6 ADORA2A Adenovirus Supplementation Can Improve the Role of AMP in Lipid Metabolism in ADORA2A Knockout Mice

**Figure 6:**
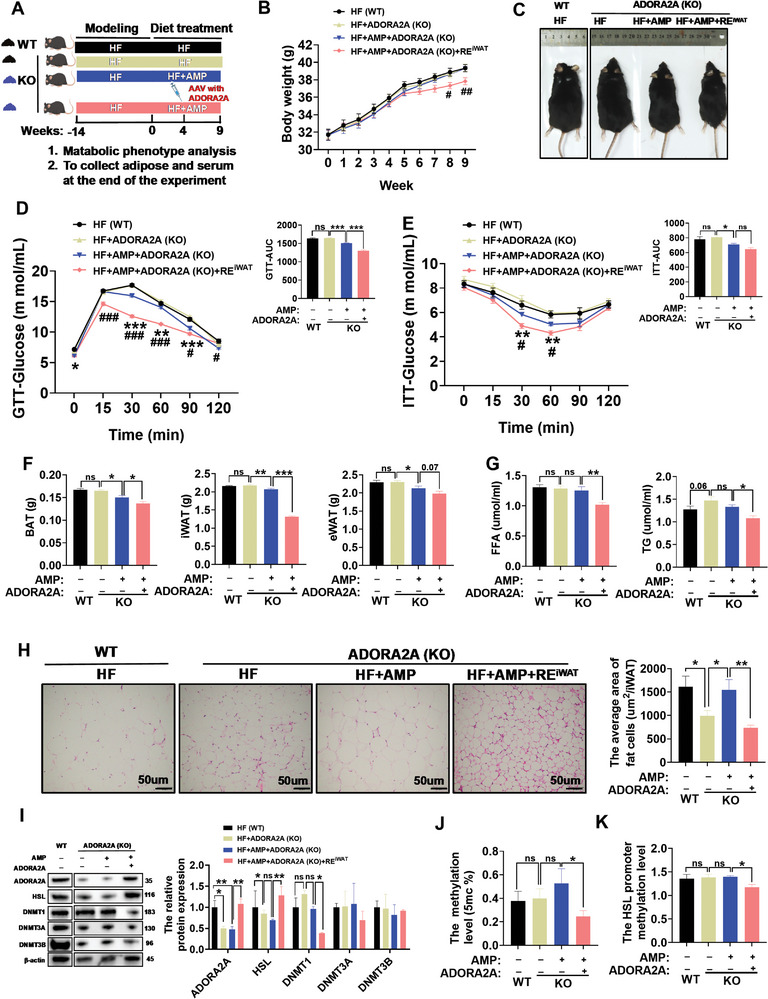
ADORA2A adenovirus supplementation can reproduce the role of AMP in lipid metabolism in ADORA2A knockout (KO) mice. A) The schematic map of adenovirus supplementation in ADORA2A KO mice. B) The change of body weight during 0–9 weeks in mice (*n* = 6–8). C) The schematic image of the somatotype of mice. D) GTT and AUC at the end of the experiment (*n* = 6‐8). After fasting overnight and then intraperitoneal injection with glucose 2 g kg^−1^, the blood glucose of the mice was measured and AUC was calculated at the specified time point (*n* = 6–8). E) ITT and AUC at the end of the experiment (*n* = 5–6). After fasting for 5 h and then injection with insulin 0.75 IU kg^−1^, the blood glucose of the mice was measured and AUC was calculated at the specified time point (*n*=5–6). F) The weight of BAT, iWAT, and eWAT in each group after slaughter (*n* = 6–8). G) The content of FFA and TG in serum (*n* = 6). H) HE staining and fat area in iWAT (*n* = 6). I) Western blotting and quantification of A2A, HSL, and DNMTs in iWAT (*n* = 3). J) Global DNA methylation level in iWAT (*n* = 6). K) The HSL promoter methylation level in iWAT(*n* = 6). Data were analyzed by one‐way ANOVA followed by post hoc Tukey's tests (Figure 6A–I) or unpaired Student's t‐test (Figure 6 J,K) and presented as mean ± SEM. In Figure 6B–I, REiWAT means targeted replacement of A2A adenovirus in white tissue. In Figure 6B–D, ^*^ and ^#^ represent the comparison of HF+ADORA2A (KO) versus HF+AMP+ADORA2A (KO), and HF+AMP+ADORA2A (KO) versus HF+AMP+ADORA2A (KO)+RE^iWAT^, respectively. ^ns^ *p* > 0.05, ^*^ *p <* 0.05, ^**^ *p <* 0.01, ^***^ *p <* 0.001, ^#^ *p <* 0.05, and ^##^ *p <* 0.01. 5mc, 5‐methylcytosine; AAV, adeno‐associated virus; AMP, adenosine monophosphate; AUC, area under curve; BAT, brown fat; DNMT1, DNA methyltransferase 1; DNMT3A, DNA methyltransferase 3A; DNMT3B, DNA methyltransferase 3B; eWAT, epididymal white adipose tissue; FFA: free fatty acid; GTT, glucose tolerance test; HE, hematoxylin‐eosin; HSL, hormone‐sensitive lipase; ITT, insulin tolerance test; iWAT, inguinal white adipose tissue; TG, triglyceride.

Whether the metabolic benefits of AMP are indeed mediated by elevated ADORA2A level was further investigated through a metabolic experiment with global knockout mice (ADORA2A KO). The experimental procedures for dietary treatment and adenovirus injection are shown in **Figure** **6**A. The knockout effect of ADORA2A and the replenishment effect of ADORA2A adenovirus were confirmed through protein and PCR analysis (Figure 6I; Figure S6A,B, Supporting Information). We found that ADORA2A adenovirus replenishment had no effect on ADORA2A level in eWAT, GAS, and SOL (Figure S6D, Supporting Information). Compared with WT mice, the ADORA2A KO mice with or without AMP supplementation showed no reduction in their body weight, in contrast to a body‐weight decrease when ADORA2A KO mice received both AMP treatment and ADORA2A adenovirus injection (Figure 6B,C). At the same time, no significant difference was observed between WT mice and KO mice in feed intake at the end of the experiment (Figure S6C). Further glucose metabolism analysis found that AMP supplementation could improve glucose tolerance and insulin resistance in ADORA2A KO mice, especially following the injection of ADORA2A adenovirus (Figure 6D,E). After ADORA2A adenovirus injection, AMP could decrease the weight of BAT and iWAT in mice but had no effect on eWAT (Figure 6F; Figure S6E, Supporting Information). Consistently, the decreased effect of AMP on serum lipid metabolism, including FFA and TG (Figure 6G), was reproduced after ADORA2A adenovirus injection in ADORA2A KO mice. Similarly, the re‐supplementation of ADORA2A adenovirus had no significant effect on the weight of muscle (Figure S6F, Supporting Information). Histopathological examination showed that the average adipocyte size of iWAT treated with ADORA2A adenovirus was decreased (Figure 6H) and the HSL protein level was reversed (Figure 6I). Furthermore, after ADORA2A adenovirus injection, we observed a decrease in DNMT1, global DNA methylation, and HSL gene methylation in KO mice treated with ADORA2A adenovirus (Figure 6I–K).

## 3 Discussion

In this study, our major finding is that AMP can prevent weight gain and improve glucose metabolism in HF‐fed mice. We observed that AMP treatment promoted the lipid decomposition of iWAT and improved glucose tolerance and insulin resistance in obese mice, but had no impact on food intake. We further provide both in vitro and in vivo evidence that the anti‐obesity effect of AMP is mediated by the transformation of CD73 into ADO. By using loss‐ and gain‐of‐function mouse model, we found that the receptor ADORA2A of ADO is essential for the stimulating effect of AMP on fat decomposition. Importantly, we demonstrated that the activation of ADORA2A can decrease DNA methylation and lead to weight loss. These findings implicate that AMP is involved in the process of anti‐obesity and has a profound effect on body metabolism as a nutrient to change the DNA methylation level.

Previous studies have revealed the effects of AMP on body weight and on BAT and skeletal muscle in mice.^[^ ^11^, ^25^ ^]^ While these findings contribute to our understanding of the role of AMP in obesity, there is still a need to elucidate the mechanism through which AMP alleviates obesity. Here we demonstrate that AMP can reverse high‐fat diet‐induced obesity phenotypes, including alterations in mouse weight, glucose metabolism, and iWAT metabolic characteristics. However, previous reports have shown that AMP supplementation in a high‐fat diet had no effect on body weight in mice.^[^ ^23^ ^]^ We speculate that this discrepancy may be due to variations in mouse models and that inducing obesity in mice prior to dietary intervention can potentiate the beneficial effect of AMP on obesity.

Obesity has been reported to be associated with the increased cycling of FFA and TG.^[^ ^26^, ^27^ ^]^ Elevated FFA levels can inhibit peripheral glucose uptake and stimulate hepatic glucose production,^[^ ^28^ ^]^ resulting in insulin resistance and impairing glucose tolerance.^[^ ^29^, ^30^ ^]^ In this study, AMP was effective in improving insulin resistance by reducing FFA levels. Triglycerides can be hydrolyzed by lipase to produce FFA,^[^ ^31^, ^32^ ^]^ which provides energy to the body through further β‐oxidation. However, the fatty acid oxidation process is reduced in obese individuals,^[^ ^33^, ^34^ ^]^ resulting in the accumulation and subsequent increase in FFA concentration in the HF group. In addition, we found that AMP increased the core temperature, interscapular surface temperature, and average energy consumption throughout the day in mice. This suggests that AMP promotes the decomposition of fat and produces FFA that may enhance the oxidation function of brown fat or liver, which may be related to the activation of AMPK or the increase in mitochondrial content,^[^ ^35^, ^36^ ^]^ thereby promoting energy release. Consistent with previous studies,^[^ ^37^, ^38^ ^]^ the increase of FFA oxidation may reduce circulating FFA levels, resulting in lower FFA levels in the AMP group compared to the HF group. The similar effects between AMP and ADO in the aforementioned metabolic phenotypes prompted us to investigate the interconversion between AMP and ADO, which can help to elucidate the mechanism of weight loss.

Under normal circumstances, extracellular ADO concentration is low and is mainly regulated by CD73.^[^ ^39^ ^]^ Our results showed that obesity did not cause changes in ADO and AMP levels in mouse fat and serum, nor did it affect the levels of proteins in the conversion between ADO and AMP. However, AMP supplementation could effectively elevate ADO levels in iWAT and serum in obese mice, while ADO treatment had no effect on AMP levels. In addition, only the CD73 protein showed a significant difference in the iWAT of ADO and AMP‐treated mice but had no effect on ADK, a key enzyme in the conversion of ADO to AMP. The above results suggest that the expression level of CD73 in iWAT is more sensitive to the changes in ADO and AMP content, suggesting that the beneficial effect of AMP dietary supplementation may depend on the transformation of CD73.

Experiments in vitro and in vivo facilitated further understanding of the role of CD73 in the anti‐lipid effect of AMP. After injection of CD73 inhibitor into the subcutaneous adipose tissue of obese mice, the effective metabolism of AMP was eliminated and the weight of iWAT increased significantly. In addition, under normal conditions, CD73 knockdown has no effect on lipid droplet formation in cells, but CD73 knockdown under conditions of AMP supplementation can eliminate the effect of AMP on lipid droplet formation, implying even in the absence of AMP, the effect of CD73 on cellular lipid metabolism will remain unchanged. Similarly, the results of AMP supplementation after blocking CD73 with drugs were consistent with the results of CD73 knockdown. This provided further evidence for the pivotal role CD73 plays in the transformation of AMP into ADO to improve lipid metabolism. In addition, we found that HSL was involved in the anti‐obesity process of AMP/CD73, because the increasing effect of AMP on HSL was significantly eliminated after CD73 knockdown, indicating that HSL is a key target for AMP/CD73 to play an anti‐obesity role.

The effect of ADO on DNA methylation has been reported. It is well documented that the increase of intracellular ADO level can reduce the promoter methylation level of vascular endothelial growth factor receptor (VEGFR) and ATP‐binding cassette subfamily G member 1 (ABCG1).^[^ ^40^, ^41^ ^]^ Therefore, AMP can be hypothesized to modulate DNA methylation following its conversion to ADO. We further found that AMP supplementation could reduce the level of DNMT1 or DNMT3A and global DNA methylation levels in iWAT, which can be antagonized by CD73 inhibitors. DNA methylation reduction is often accompanied by an increase in the expression of some genes,^[^ ^42^ ^]^ which may be related to the increase in HSL level. Despite no CpG island in the HSL promoter based on our prediction through the Methpriimer online website, this study revealed that AMP reduced HSL promoter methylation in iWAT in DIO mice, with a substantial proportion also occurring at non‐CpG sites.^[^ ^43^ ^]^ To further explore the connection between AMP and DNA methylation, we specifically used DNA methylation inhibitors to treat cells. Interestingly, AMP treatment showed consistent cell phenotypes after treatment with DNA methylase inhibitors, and both of them reduced HSL promoter methylation level. Consistently, more accurate bisulfite sequencing analysis revealed that AMP did reduce HSL promoter methylation. However, after CD73 knockdown CD73, the HSL methylation level was restored. Overall, these findings suggest that the DNA methylation mediated by the conversion of AMP to ADO may be a potential target for reducing obesity and its related complications.

The role of ADO receptors in DNA methylation is still controversial. It is well known that ADO can be transferred into cells through nucleoside transporters, or it can bind to receptors to play a role.^[^ ^44^ ^]^ By nucleoside transporter knockdown, we found that the beneficial effects of AMP cannot be completely reversed. Therefore, the conversion of AMP to ADO may be achieved through adenosine receptor activation by CD73 to promote the binding between ADO and its receptors. To further study the effect of AMP on DNA methylation after its conversion to ADO, we detected the expression levels of different ADO receptors. Previous studies have shown that the increase of intracellular ADO can promote DNA hypomethylation in the placenta of preeclampsia, and this effect is not related to ADORA2B signal transduction.^[^ ^45^ ^]^ However, ADORA1 knockout was found to reduce DNA methylation in embryonic mice.^[^ ^46^ ^]^ In this study, we observed higher ADORA2A and lower ADORA3 in iWAT of obese mice treated with AMP and ADO versus the control mice. The in vitro test found that ADORA2A inhibition or ADORA3 activation could eliminate the inhibitory effect of ADO on the formation of lipid droplets. Notably, the decreasing effect of ADO on global DNA methylation and HSL gene methylation levels was eliminated only when ADORA2A was inhibited. By further ADORA2A knockdown, we could also obtain the same effect as ADORA2A inhibitors, while ADORA3 overexpression had no effect on DNA methylases. Therefore, we tested whether the regulation of DNA methylation by AMP is mediated by ADORA2A in knockout mice. Compared with the ADORA2A KO mice without adenovirus injection, the ADORA2A KO mice supplemented with adipose tissue‐targeted ADORA2A adenovirus showed increased sensitivity to AMP dietary intervention, with significant improvement in insulin resistance and obesity. Note that AMP dietary intervention still has a certain degree of effectiveness in improving insulin resistance and obesity after ADORA2A KO, probably because AMP can also impact physiological processes through alternative pathways. Meanwhile, it is undeniable that ADORA2A partially plays a role in mediating the effects of AMP, as ADORA2A adenovirus injection can re‐reduce the levels of DNA methylation and HSL methylation in ADORA2A knockout mice supplemented with AMP. Our results suggest that AMP can affect HSL promoter methylation at least partly through ADORA2A and improve the process of lipid metabolism.

There are several limitations to the present study. First, AMP has effects on both iWAT and BAT, but we have not further verified the relationship between lipolysis in iWAT and energy consumption in BAT. Secondly, the anti‐obesity effect of AMP is a complex process that involves many pathways. However, the current study only focused on the role of DNA methylation in the anti‐obesity effect of AMP without exploration of other pathways. Finally, we only studied the correlation between the lipidity‐lowering function of AMP and the methylation level of HSL and did not further explore how DNA methylase affects the methylation of HSL promoter. In further studies, we intend to gradually address these questions to further understand the mechanistic links between AMP and obesity.

**Figure 7:**
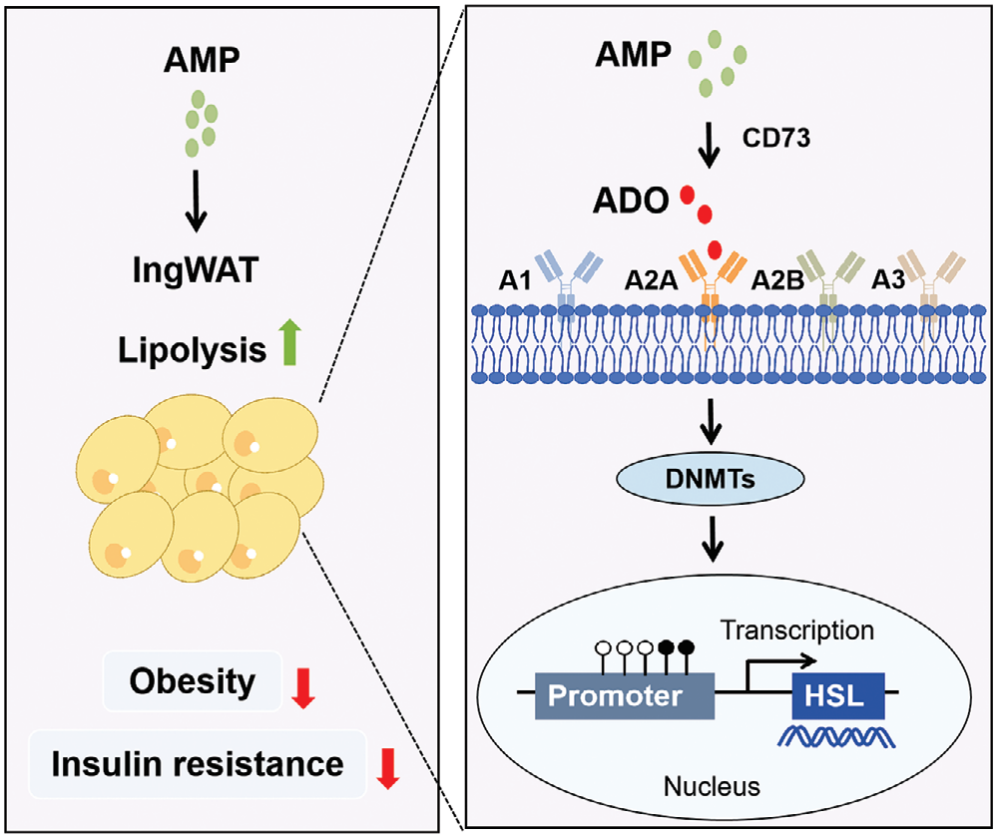
The mechanism diagram for the protective effects of AMP‐mediated adenosine signaling pathway on hyperlipidemic obese mice. AMP, adenosine monophosphate; ADO, adenosine; A2A, adenosine receptor 2A; A1, adenosine receptor 1; A3, adenosine receptor 3; A2B, adenosine receptor 2B; CD73, ecto‐5′‐nucleotidase; DNMTs, DNA methyltransferase; HSL, hormone‐sensitive lipase.

In summary, as shown in **Figure** **7**, the improvement effect of AMP on obesity can be attributed to the conversion of extracellular AMP to ADO through ADORA2A activation by CD73, which can promote lipolysis by reducing DNA methylation level and enhancing HSL gene transcription. These findings show the great potential of AMP in improving obesity.

## 4 Experimental Section

### Obese Mice and AMP Treatment

The animal research was conducted in accordance with the guidelines for the Review of Experimental Animal Welfare and Ethics in Guangdong Province and approved by the Ethical Committee for Animal Care and Utilization of Southern China Agricultural University (2022F233). C57BL/6 mice were purchased from Guangdong Animal Experimental Center (Guangzhou City, Guangdong Province, China) and were housed under controlled conditions of temperature and humidity (23 ± 3 °C/70 ± 10%), a 12 h light–dark cycle, and ad libitum access to food and water. All mice underwent a two‐week acclimation period in a standardized environment before any experimental procedures commenced. Male mice of similar age were randomly allocated into different experimental groups based on their body weight.

### Construction of Obese Mice

The forty‐four 5‐week‐old mice were randomly divided into two groups based on consistent body weight. One group of 11 mice was fed a basic diet (Jiangsu Collaborative Medical and Biological Engineering, carbohydrate content: 70%, protein content: 20%, fat content: 10%), while the other group of 33 mice was fed a high‐fat diet (Jiangsu Collaborative Medical Bioengineering, carbohydrate content: 20%, protein content: 20%, fat content: 60%) to form obese mice. After 10 weeks, the obese mice were successfully established. Following the consistent body weight principle, the obese mice were then randomly divided into three groups for 16 weeks: high‐fat diet group (HF), high‐fat diet supplemented with 0.1% ADO group (HF+ADO), and high‐fat diet supplemented with 0.1% AMP group (HF+AMP), with 11 mice in each group. The control group (CON) continued to receive the basic diet throughout the 16‐week experimental period. During the experiment, the feed intake was measured every 3 days, and the body weight was measured once a week. At the end of the experiment, the glucose tolerance test (GTT) and insulin tolerance test (ITT) were conducted in mice. After dissection, the fixed and molecular samples of inguinal white adipose tissue (iWAT), brown fat (BAT), epididymal white adipose tissue (eWAT), and muscle were collected for further analysis.

In another mouse experiment, twenty‐four 5‐week‐old male mice were treated with a high‐fat diet for 14 weeks to induce obese mice, and then randomly assigned into three groups: high‐fat diet group (HF), a high‐fat diet supplemented with 0.1% AMP group (HF+AMP), and a high‐fat diet supplemented with 0.1% AMP group and treated with CD73 inhibitor (HF+AMP+PSB‐12379). PSB‐12379 (30 mg kg^−1^) was injected into the iWAT in the 4th week after the successful construction of obese mice, followed by GTT and ITT and collecting samples for further analysis at 5 weeks post‐injection.

### ADORA2A Knockout (KO) Mouse Model

The ADORA2A KO mouse model was established by Guangdong Yaokang Biotechnology Co., Ltd. There were four transcripts of ADORA2A gene, and the exon2‐exon3 of ADORA2A‐201 (ENSMUST00000105420.2) was targeted for knockout due to its containing all coding sequences essential for protein function. F0 and F1 mice were genotyped by PCR using the following pair of primers: forward: 5′‐GGTAGTATCCTGAAGGGGGAGC‐3′; reverse: 5′‐ TAGCAGTGTCTCCTCGATGGAGG‐3′. The positive heterozygous F0 genotype was confirmed by sequencing, and the heterozygous F0 mice were selected and bred to produce F1‐positive genotypes. Genotypes of both F0 and F1 mice were identified by PCR and confirmed by sequencing, followed by the comparison of ADORA2A mRNA expression between wild‐type and ADORA2A KO mice.

To confirm the anti‐obesity role of ADORA2A in AMP, 5‐week‐old wild‐type (WT) mice and ADORA2A knockout (KO) mice were subjected to a high‐fat diet for 14 weeks. Subsequently, the ADORA2A KO mice were randomly divided into three groups: one group continued to receive the high‐fat diet, while the other two groups received supplementation with an AMP diet, with one group receiving local injection of adeno‐associated virus (AAV) carrying ADORA2A into iWAT after 4 weeks of AMP supplementation. ADORA2A AAV was constructed by Hanheng Biotechnology (Shanghai) Co., Ltd., using the AdipoQ promoter to induce adipocyte‐specific adeno‐associated virus 2max 9‐mediated FGF6 or GFP overexpression (1.6×10^12^ vg mL^−1^). The injection process of adenovirus is as follows: 1) Mice were anesthetized with 5% isoflurane and eye ointment was applied to prevent dryness. 2) The skin at the groin area was depilated and cleaned with alcohol. 3) A small incision was made with a scalpel and then gently separated to expose iWAT. 4) The virus (50 µL) was injected into iWAT at multiple spots on each side. 5) Finally, the fat pad was placed back into position and sutured. GTT, ITT, and dissection procedures were conducted at five weeks post‐adenovirus injection to collect fixed and molecular samples from fat and muscle tissues for further analysis.

### Cold Exposure Treatment and Body Composition Detection

Six mice per group were randomly selected for body temperature measurement using a brown fat‐12 microprobe thermometer (Physitemp, USA). Baseline temperatures were recorded at 0 h before mice exposure to acute cold in a 4 °C incubator for 6 h. Throughout the experiment, the mice drank water freely and their body temperature was measured once every 1 h. Post‐exposure BAT temperature was assessed using infrared detectors and thermal images were captured for analysis with FLIR Tools. At week 12 of the study, eight mice were randomly selected from each group for assessment of body composition (including adipose tissue content and lean muscle mass) utilizing an EchoMRI 3‐in‐1 quantitative magnetic resonance (QMR) composition analyzer (Echo Medical Systems, Houston, TX).

### Energy Metabolism Measurement

At week 13, six mice were randomly selected from each group and placed into a Promethion mouse metabolic cage tester (Sable Systems International) with ad libitum access to water and food throughout the experiment, and the assay was initiated following a 24 h acclimation period. The minimum, maximum, and average energy consumption were recorded for all the mice.

### Glucose tolerance test (GTT)/Insulin tolerance test (ITT) Detection

GTT and ITT were performed at a designated time. For GTT, mice were fasted overnight (12 h) before being administered glucose (2 g kg^−1^ body weight), and blood glucose levels were monitored at various time intervals. For ITT, mice fasted for 5 h and received a single dose of insulin (0.75 IU kg^−1^ body weight), followed by measuring the blood glucose levels in the tail vein using a Sinocare blood glucose meter.

### Free Fatty Acid (FFA)/Triglyceride (TG) Detection

According to the manufacturer's instructions (Nanjing Built Biological Engineering Research Institute, Nanjing, China), the TG kit was used to determine the TG content in mouse serum, and the FFA determination kit was used to determine the FFA content in mouse serum.

### Gene Knockdown in 3T3‐L1 Cell

The solute carrier family 29 member 1 (ENT1), CD73, and ADORA2A siRNA plasmid were purchased from Jima Gene Co., Ltd. (Suzhou, China) and transfected into 3T3‐L1 cells using as instructed by the manufacturer (Thermo Fisher Technology Co., Ltd., China). The sequences of siRNA targeting ENT1 are 5′‐GCAACCAAGUAUUUCACAATT‐3′ (sense) and 5′ ‐UUGUGAAAUACUUGGUUGCTT‐3′ (anti‐sense). The sequences of siRNA targeting CD73 are 5′‐GCAGCCUGAAGUAGAUAAATT‐3′ (sense) and 5′ ‐UUUAUCUACUUCAGGCUGCTT‐3′ (anti‐sense). The sequences of siRNA targeting ADORA2A are 5′‐CCCAUGAAUUACAUGGUUUTT‐3′ (sense) and 5′ ‐AAACCAUGUAAUUCAUGGGTT‐3′ (anti‐sense). The si‐RNA plasmid were transfected during the second induction of differentiation in 3T3 cells and the third replacement with a complete medium, and the medium was completely replaced at 6 h post‐transfection.

### qPCR

The real‐time quantitative PCR measurement was conducted as previously described.^[^ ^47^ ^]^ Briefly, total RNA was extracted from adipose tissue using the Tissue RNA Purification Kit (for Adipose Tissue) (EZBioscience, Suzhou Yingze organism). Then total mRNA was reverse transcribed into cDNA using the Color Reverse Transcription Kit (EZBioscience, Suzhou Yingze organism). Finally, qPCR was performed on an ABI QuantStudioTM 6 Flex System (Applied Bioscience, Carlsbad, CA) using the iScrip one‐step RT‐PCR kit with SYBR Green (A0002/EZ Bioscience, China), and the expression of the housekeeping gene β‐actin was used for normalization of results. The primers used are shown in Table S2, Supporting Information.

### Western Blotting (WB)

WB analysis was conducted following standard procedures. The white adipose tissue (≈−100 mg) was dissolved in the proteolysis solution containing protease and phosphatase inhibitors (P1048dBeyotime, China). Subsequently, proteins were transferred onto polyvinylidene fluoride membranes and probed with primary and secondary antibodies (Table S3, Supporting Information). The proteins were visualized using a chemiluminescence reagent (P10200–NCM Biotech, China), followed by quantification of signal intensity on the film using Image Lab software.

### Hematoxylin‐eosin (HE) Staining

Six iWAT and BAT slice samples from each group of mice were randomly selected and dispatched to Biossci (Hubei) Biotechnologies Company Limited (Wuhan, China). The samples were sectioned along the median sagittal plane, followed by paraffin embedding and HE staining. Finally, the size of BAT lipid droplets was assessed using a microscope, and the average cell area of iWAT was calculated.

### Liquid Chromatogram‐Mass Spectrometry (LC‐MS)

The levels of ADO, AMP, uric acid, adenine, xanthine, and hypoxanthine in serum and iWAT were quantified using *LC‐MS*. Briefly, the methanol (800 µL) was added to a homogenate containing serum (200 µL) or tissue (200 µL), followed by vortexing the mixture for 2 min and centrifugation at 4 °C for 15 min. Next, the resulting supernatant (1000 µL) was transferred to a centrifuge tube, followed by vacuum‐centrifugation for 2 h and then nitrogen drying at room temperature. After desiccation, the sample was reconstituted in a solution containing 50% methanol‐water (200 µL), followed by vortexing for another 2 min, ultrasonication in an ice bath for 10 min, and then centrifugation at 14 500 rpm at 4 °C for another 15 min. Finally, the resulting supernatant was transferred to an injection vial (with village tube) and stored at −80 °C for further analysis.

### Determination of Lipid Droplets

The 3T3‐L1 cells were induced to differentiate at 37 °C and 5% CO_2_, coupled with the supplementation of ADO (Sigma, Germany), AMP (Sigma, Germany), 5‐Aza‐Cdr (sigma, Germany), CD73 inhibitor (Sigma, Germany), ADORA2A inhibitor (Sigma, Germany) or ADORA3 agonist (Sigma, Germany) during the differentiation process. After 6 days of treatment, the supernatant was discarded and the cells were collected for further analysis.

For the determination of lipid droplets, the cell culture medium was discarded and phosphate buffer saline (PBS) was used for cell washing. Subsequently, the cells were fixed with 4% paraformaldehyde at room temperature for 10 min, followed by two rinses with PBS and then adding a small amount of 60% isopropanol to cover the cells for 20 s. After absorption and drying, an oil‐red O staining solution was applied to cover the cells in an opaque container in the darkness at room temperature for 30 min. Finally, rapid differentiation with 60% isopropanol was performed for 3–5 s, followed by three 5 min washes with pure water, then adding PBS to cover the cells and observation under a microscope.

### Detection of Global Methylation Level

The DNA digestion method was slightly modified based on Huang et al.^[^ ^48^ ^]^ Briefly, total tissue DNA was extracted using the Bio Flux DNA extraction kit as instructed by the manufacturer. After dilution (50 ng µL^−1^), the DNA (2.5 µg) was placed in a centrifuge tube (50 µL), followed by adding 70% perchloric acid (25 µL) and hydrolyzing the mixture at 80 °C for 5 h with diethypyrocarbonate (DEPC) water. Subsequently, the hydrogen ion concentration (pH) value was adjusted to 3–5 using potassium hydroxide (KOH) solution (diluted by 100 times), resulting in precipitation of potassium perchlorate (KClO4) and then standing overnight in a ‐20 °C refrigerator. After centrifugation (12 000 rpm, 30 min), the supernatant was stored in the refrigerator overnight to precipitate again. After another centrifugation (12 000 rpm, 30 min), the supernatant was transferred into sample bottles for detection. The global DNA methylation level was expressed as a percentage of cytosine methylation to total cytosine content.^[^ ^49^ ^]^

### Methylation‐Specific PCR (MSP)

DNA extraction and bisulfite conversion followed the previous methods.^[^ ^50^ ^]^ Briefly, total tissue DNA (BSC04M1, Biospin) was extracted using the Bio Flux DNA extraction kit as instructed by the manufacturer and then converted into bisulfite following the protocol of the EZ DNA methylation kit‐GOLD kit (D5005 Zymo). The CT conversion (130 µL) reagent was added to a DNA sample (20 µL) with a total input of 500 ng, followed by subjecting the sample tube to thermal cycling (98 °C for 10 min and 64 °C for 2.5 h), and then purification using a Zymo‐Spin IC column with M‐Binding Buffer. Methylation‐specific primers (Table S4, Supporting Information) were designed and synthesized by Shanghai Shengong Bioengineering Co., Ltd. (Shanghai, China), and used for PCR amplification of bisulfite‐treated genomic DNA.

### Bisulfite Sequencing PCR (BSP)

DNA was extracted according to the kit protocol, and the extracted DNA was treated with bisulfite and then subjected to PCR amplification. Next, the PCR products were soaked in 5 µL  % agarose gel electrophoresis, followed by electrophoretic observation 10–20 min later, cutting off the target strip with a scalpel and recovering the products according to the kit protocol. Finally, the recovered PCR products were subjected to cloning and sequencing in the following steps: a) 100 µL of competent cells were placed on ice, followed by gently suspending them evenly after completely thawing. b) 10 µL of connection solution was added and gently well mixed with the cells, followed by standing on ice for 30 min. c) The mixture was heat shocked in a 42 °C water bath for 45 s and then left on ice for 15–20 min. d) The mixture was supplemented with 600 µL of SOC medium and cultured at 37 °C under shaking at 200–250 rpm for 1 h. e) After centrifugation at 4000 rpm for 5 min at room temperature, use the gun tip was used to suck off 400 µL of supernatant, and use the remaining medium was used to suspend the cells. f) Bacteria were spread on ampicillin plates previously coated with 20 µL of 100 mm IPTG and 100 µL of 20 mg mL^−1^ X‐gal. g) The plate was cultured forward for 1 h at 37 °C to absorb excess liquid, followed by incubation upside down overnight, and then single colonies were selected for sequencing. The first 3 kb position of the HSL promoter site was selected for sequencing and finally determined it as a 1270–1631 bp fragment, with the sequence including 6 CG sites:

CCGGCTGCACTTGCCACTCTTGTCTTATGCCTTCTGTTTACCCTACAAGAATGCAGGACACCTCGGCCTCCTGTTGGCCTCACATTGCAATCAGAAAACTTGCAATGAAAATAATTAAATACATAGTGCATACGTGTGTGTATATGTGTGTATGTAAAGTGAGTATCAGTTATATCGATCAGATGGGGTCTGACTACTGACATTCAGTTCCTGTGTCACCCTTAGCCCTGTTCATTTGAAACCCTTCCCATGTACTTCTCCCTCCTTGCCCTTCTCTACTCTTTCACTCGTTTGGAGGTGCACGGTCT

### Data Analysis

The data were analyzed using SPSS 22.0 software and plotted with GraphPad Prism 8.0 software. The results were all expressed as mean ± standard error (variance ± SEM), *p* < 0.05 indicated a significant difference, 0.05 < *p* ≤ 0.10 indicated a changing trend, and *p* > 0.10 indicated no significant difference.

## Conflict of Interest

The authors declare no conflict of interest.

## Author Contributions

Z.C., L.F., and S.R. have contributed equally to this work. C.Q.T., Z.J.C., and J.P.D. performed the conceptualization. C.Q.T., Z.J.C., L.F., and S.J.R. curated data. C.Q.T. and Z.J.C developed the methodology. Z.H.H., S.B.H., L.D.L., Y.L., Z.L., Q.L.C., and L.L.W. performed the investigation. C.Q.T. visualized the study. Z.J.C. wrote the original draft. C.Q.T. wrote, reviewed, and edited the final draft. J.P.D. and Y.L.Y. administered the project.

## Supporting information



Supporting Information

## Data Availability

All data needed to evaluate the conclusions in the paper are presented in the paper and/or the Supplementary Materials. Additional data related to this paper may be requested from the authors.
