# Supplementary material for: Adenosine Monophosphate Improves Lipolysis in Obese Mice by Reducing DNA Methylation via ADORA2A Activation by Ecto‐5′‐Nucleotidase (CD73)
Source: Adv Sci (Weinh). 2025 Feb 20;12(14):2405079. doi: 10.1002/advs.202405079 (PMC11984851; doi:10.1002/advs.202405079)
Supplement: Supplementary file 1 — Supporting Information [file ADVS-12-2405079-s001.docx]

Supporting Information

Adenosine Monophosphate Improves Lipolysis in Obese Mice by Reducing DNA Methylation via ADORA2A Mediation Activation by Ecto-5'-Nucleotidase (CD73)

*Zhijuan Cui^#^*, *Li Feng^#^*, *Sujuan Rao^#^, Zihao Huang, Shuangbo Huang, Liudan Liu*, *Yuan Liao, Zheng Lan, Qiling Chen*, *Jinping Deng, Yulong Yin, Chengquan Tan**

Figure S1


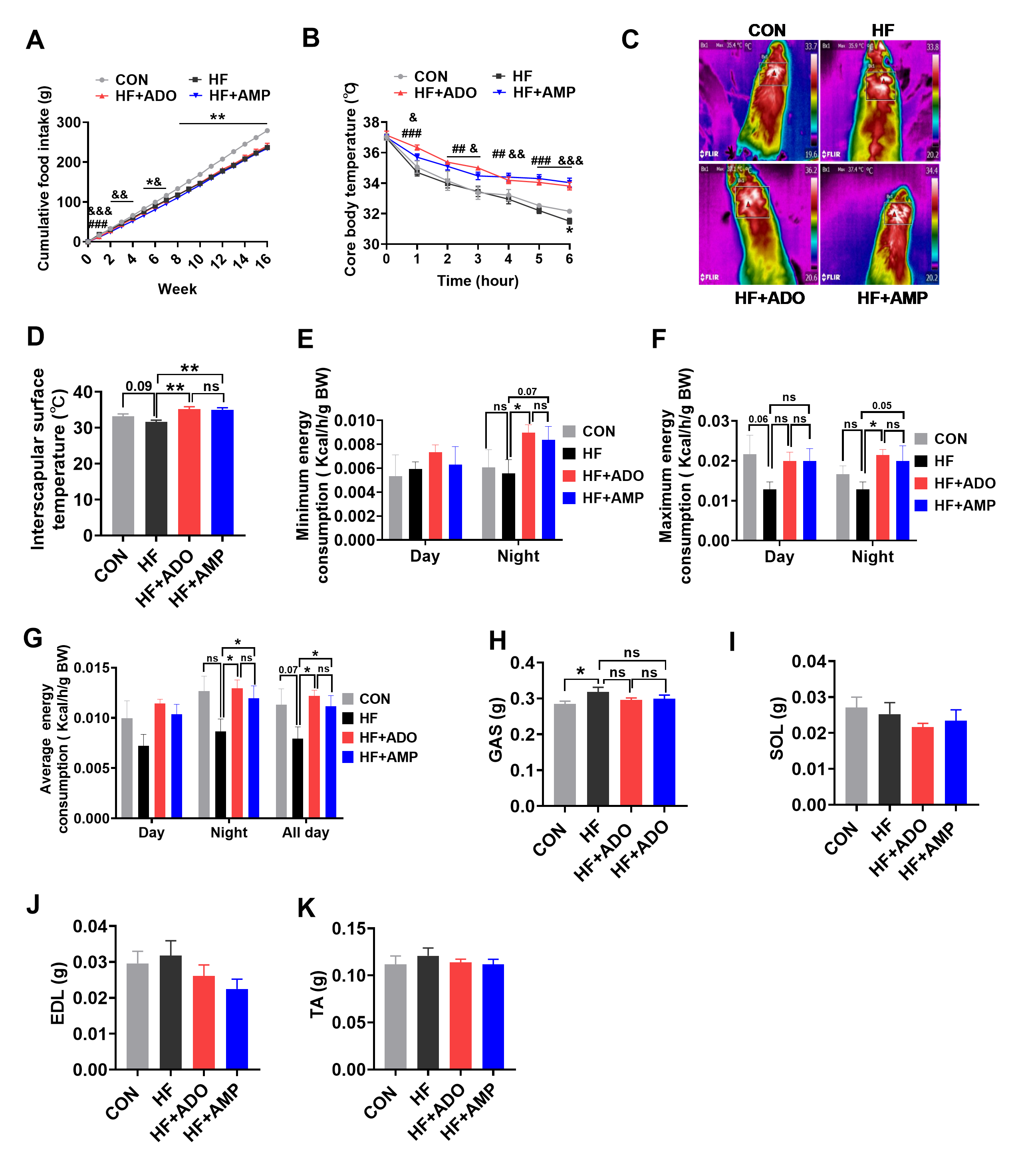


Figure S1. Exogenous AMP supplementation improves the metabolic phenotype of obese mice (A) The accumulated feed intake within 0-16 weeks (*n* = 11). (B) The trend of rectal temperature under cold treatment (*n* = 7-8). (C) The thermography of mice under cold-treatment (*n* = 6). (D) The BAT average temperature (*n* = 8). (E) The minimum energy consumption of mice (*n* = 4-7). (F) The maximum energy consumption of mice (*n* = 6-7). (G) The average energy consumption of mice (*n* = 5-7). (H-K) The weight of GAS, SOL, EDL, and TA in mice (*n* = 8), respectively. ^*^, ^#^ and ^&^ represent the comparison of HF versus CON, HF+ADO, and HF+AMP, respectively. Data were analyzed by one-way ANOVA followed by post hoc Tukey’s tests and presented as mean ± SEM. ^*^*p* < 0.05, ^#^*p* < 0.05, and ^&^*p* < 0.05. ADO, adenosine; AMP, adenosine monophosphate; BAT, brown fat; EDL, extensor digitorum longus; GAS, gastrocnemius muscle weight; SOL, soleus muscle; TA, tibialis anterior muscle.

Figure S2


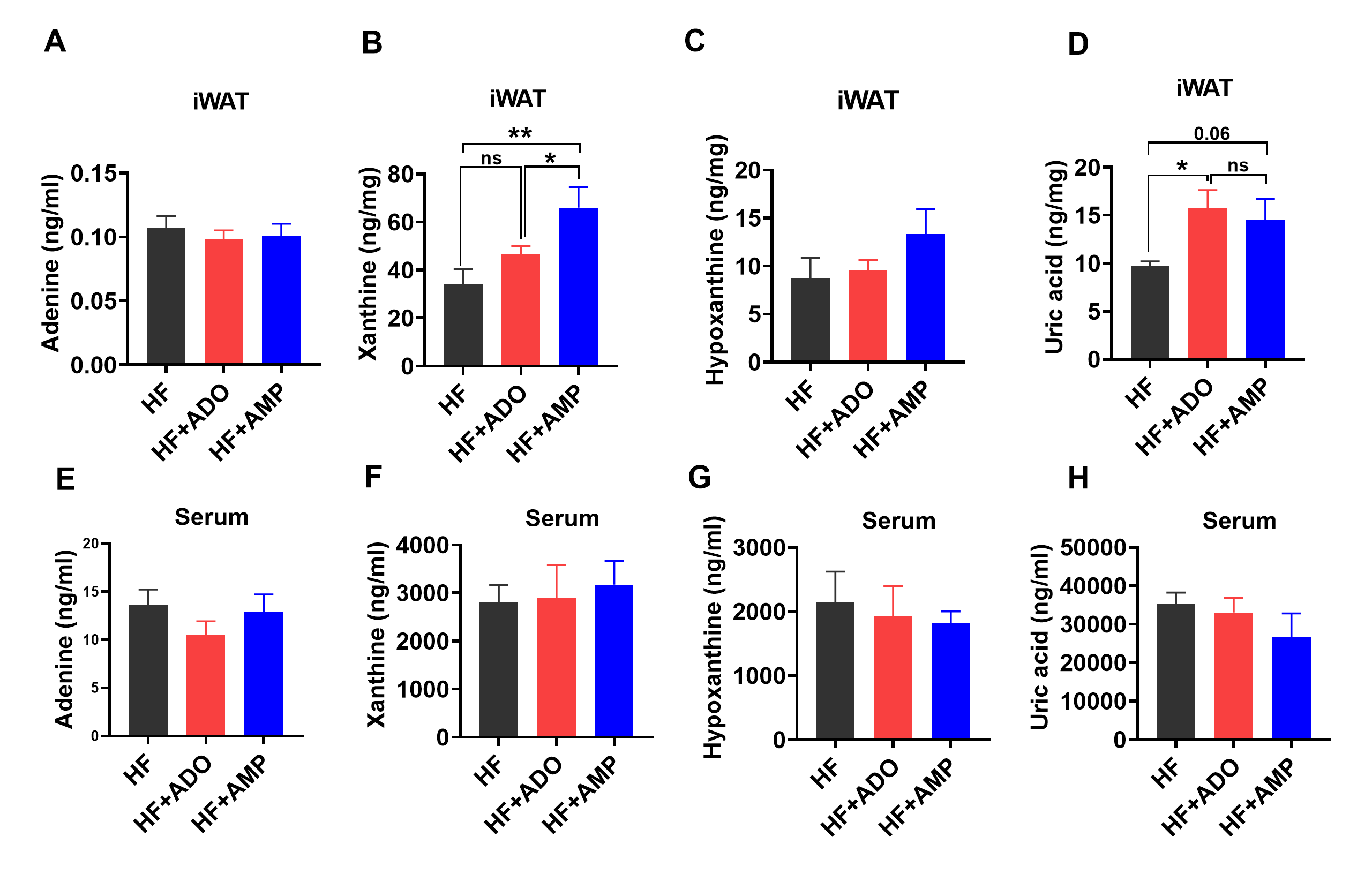


Figure S2. CD73 promotes transformation of AMP into ADO. (A-D) The content of adenine, xanthine, hypoxanthine and uric acid in iWAT (*n* = 6). (E-H) The content of adenine, xanthine, hypoxanthine and uric acid in serum (*n* = 6), respectively. Data were analyzed by one-way ANOVA followed by post hoc Tukey’s tests and presented as mean ± SEM. ^ns^*p* > 0.05, ^*^*p* < 0.05, and ^**^*p* < 0.01. ADO, adenosine; AMP, adenosine monophosphate; iWAT, inguinal white adipose tissue.

Figure S3


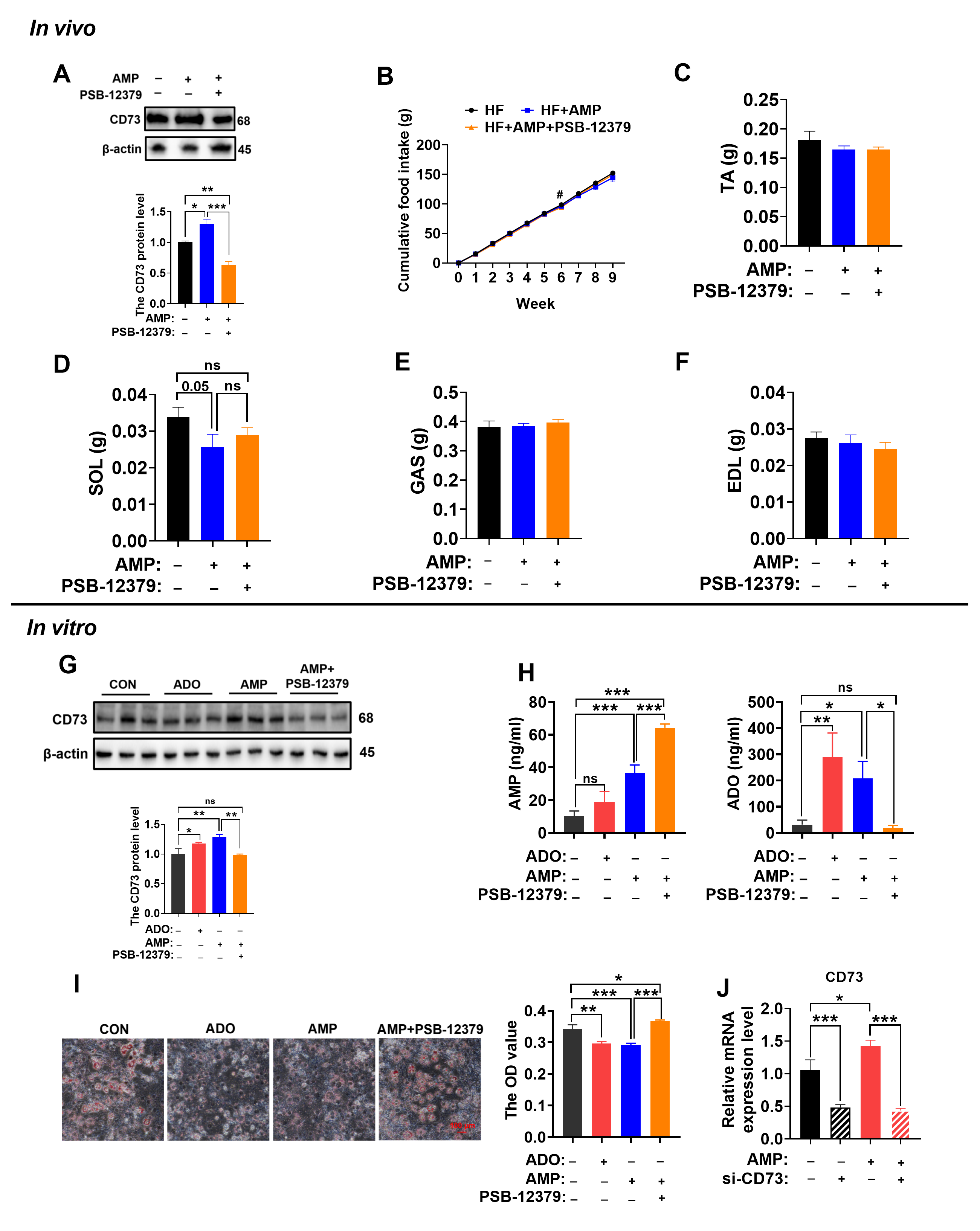


**Figure S3**. AMP is metabolized into ADO by CD73 to play an anti-obesity role. (A) Western blotting and quantification of CD73 in iWAT after injection of CD73 inhibitor (30 mg/kg PSB-12379) (*n* = 3). (B) The accumulated feed intake of mice within 0-16 weeks (*n* = 6-8). (C-F) The weight of GAS, SOL, EDL, and TA in mice after injection of CD73 inhibitor (30 mg/kg PSB-12379) (*n* = 6-7), respectively. (G) Western blotting and quantification of CD73 in 3T3-L1 cells after adding 1μm ADO, 1μm AMP and CD73 inhibitors (1μm PSB-12379) (*n* = 3), respectively. (H) The content of AMP and ADO in 3T3-L1 cells after adding 1μm ADO, 1μm AMP and CD73 inhibitors (1μm PSB-12379), respectively (*n* = 6). (I) Lipid droplet production and statistics of 3T3-L1 cells after adding 1μm ADO, 1μm AMP and CD73 inhibitors (1μm PSB-12379), respectively (*n* = 5), respectively. (J) Real-time fluorescence quantification of CD73 in 3T3-L1 cells after CD73 knockdown (*n* = 6). Data were analyzed by one-way ANOVA followed by post hoc Tukey’s tests and presented as mean ± SEM. In **SFigure** 3B, ^*^, ^&^ and ^#^ represents the comparison of HF versus AMP, HF+AMP versus HF+AMP+PSB-12379, and HF versus HF+AMP+PSB-12379 respectively. ^ns^*p* > 0.05, ^*^*p* < 0.05, ^**^*p* < 0.01, and ^***^*p* < 0.001. ADO, adenosine; AMP, adenosine monophosphate; CD73, ecto-5'-nucleotidase.

Figure S4


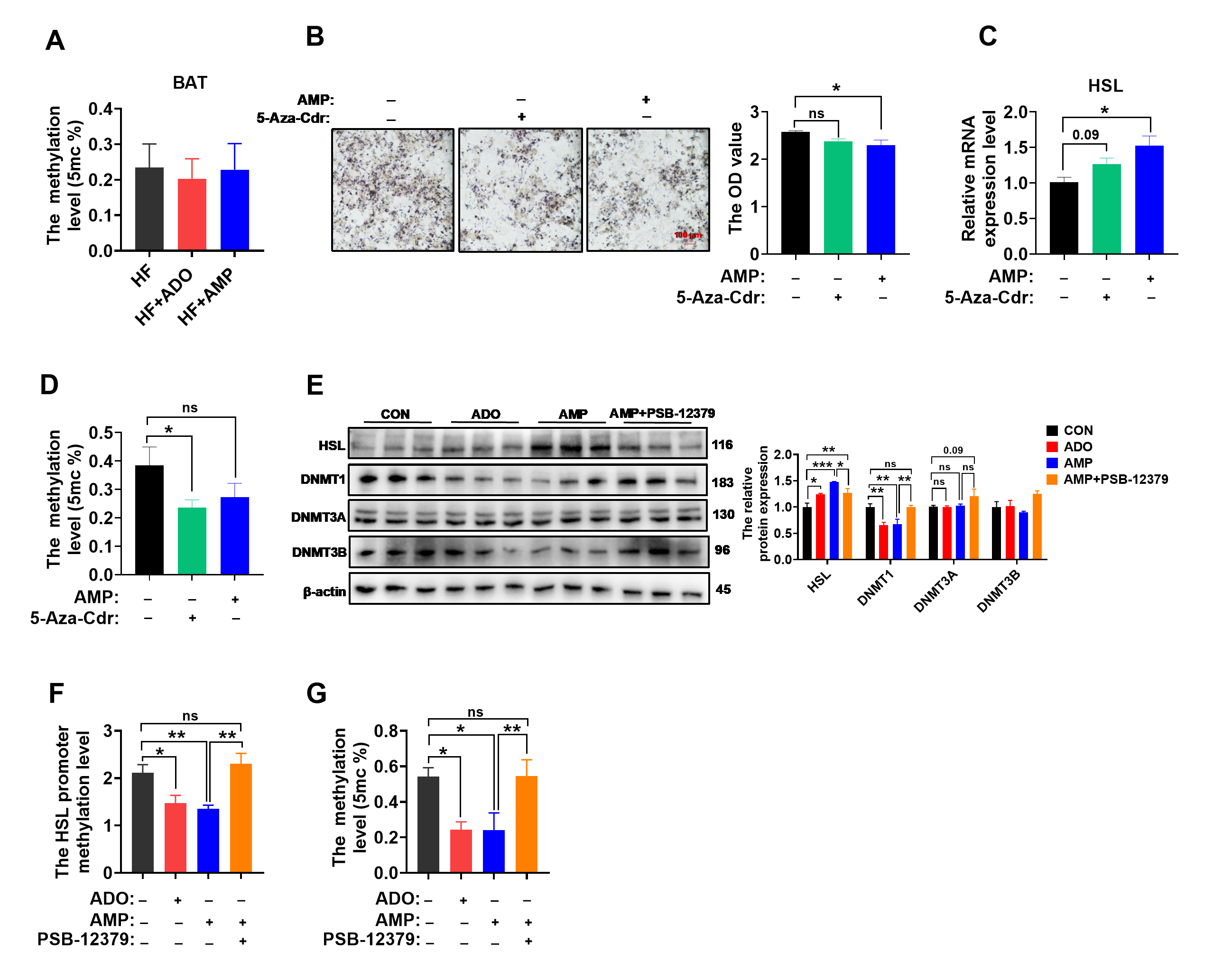


Figure S4. AMP elevates HSL levels by reducing DNA methylation through CD73 expression. (A) The global DNA methylation level in BAT (*n* = 6). (B) Lipid droplet production and statistics of 3T3-L1 cells after adding 10 μm 5-Aza-Cdr and1μm AMP respectively (*n* = 3). (C) Real-time fluorescence quantification of HSL in 3T3-L1 cells after adding 10 μm 5-Aza-Cdr and 1μm AMP (*n* = 6), respectively. (D) The global DNA methylation level in iWAT after injection of CD73 inhibitor (30 mg/kg PSB-12379) (*n* = 6). (E) Western blotting and quantification of DNMTs and HSL in 3T3-L1 cells after adding 1μm ADO, 1μm AMP and CD73 inhibitors (1μm PSB-12379) (*n* = 3), respectively. (F) The HSL methylation level in iWAT after injection of CD73 inhibitor (30 mg/kg PSB-12379) (*n* = 6), respectively. (G) The global DNA methylation level in in 3T3-L1 cells after adding 1μm ADO, 1μm AMP and CD73 inhibitors (1μm PSB-12379), respectively (*n* = 6). Data were analyzed by one-way ANOVA followed by post hoc Tukey’s tests and presented as mean ± SEM (Figure S4 A-F) or unpaired Student’s t-test (SFig 4 G)., ^ns^*p* > 0.05, and ^*^*p* < 0.05. BAT, brown fat; iWAT, inguinal white adipose tissue; 5mc, 5-methylcytosine.

Figure S5


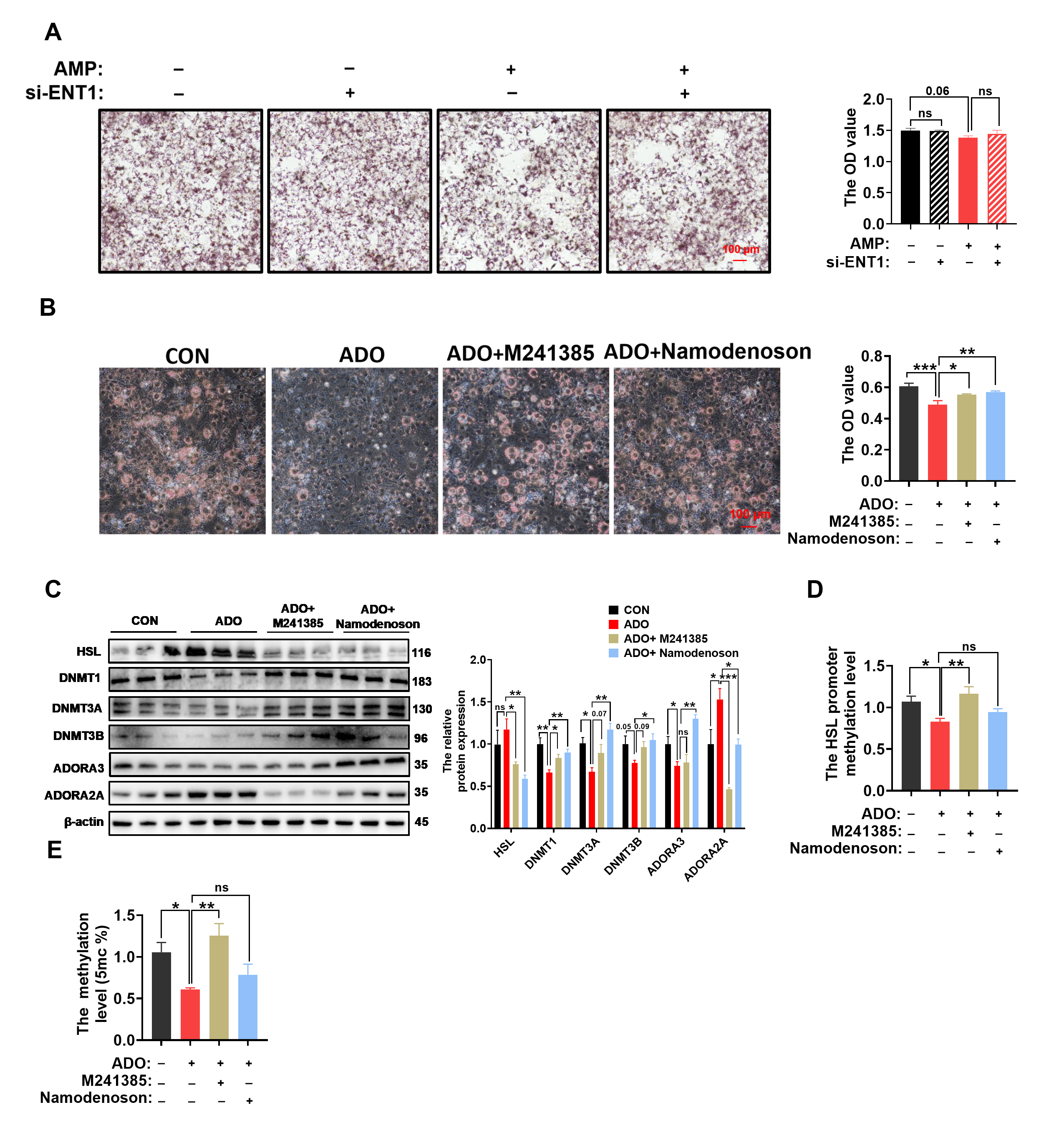


Figure S5. ADORA2A is involved in HSL promoter methylation reduction by AMP. (A) Lipid droplet formation and statistics of 3T3-L1 cells after ENT1 knockdown(*n* = 3). (B) Lipid droplet formation and statistics of 3T3-L1 cells after adding ADORA2A inhibitor (10 μm M241385) and ADORA3 agonist (10 μm Namodenoson) in vitro (*n* = 6). (C) Western blotting and quantification of ADORA2A, ADORA3, HSL, and DNMTs in 3T3-L1 cell after adding ADORA2A inhibitor (10 μm M241385) and ADORA3 agonist (10 μm Namodenoson) in vitro (*n* = 3), respectively. (D) HSL methylation level in 3T3-L1 cells after adding ADORA2A inhibitor (10 μm M241385) and ADORA3 (10 μm Namodenoson) agonist in vitro (*n* = 6), respectively. (E) Global DNA methylation level in 3T3-L1 cells after adding ADORA2A inhibitor (10 μm M241385) and ADORA3 (10 μm Namodenoson) agonist in vitro (*n* = 4), respectively. Data were analyzed by one-way ANOVA followed by post hoc Tukey’s tests and presented as mean ± SEM. ^ns^*p* > 0.05, ^*^*p* < 0.05, and ^**^*p* < 0.01. 5mc, 5-methylcytosine; ADO, adenosine; AMP, adenosine monophosphate; ADO, adenosine; DNMT1, DNA methyltransferase 1; DNMT3A, DNA methyltransferase 3A; DNMT3B, DNA methyltransferase 3B; HSL, hormone-sensitive lipase; iWAT, inguinal white adipose tissue.

Figure S6


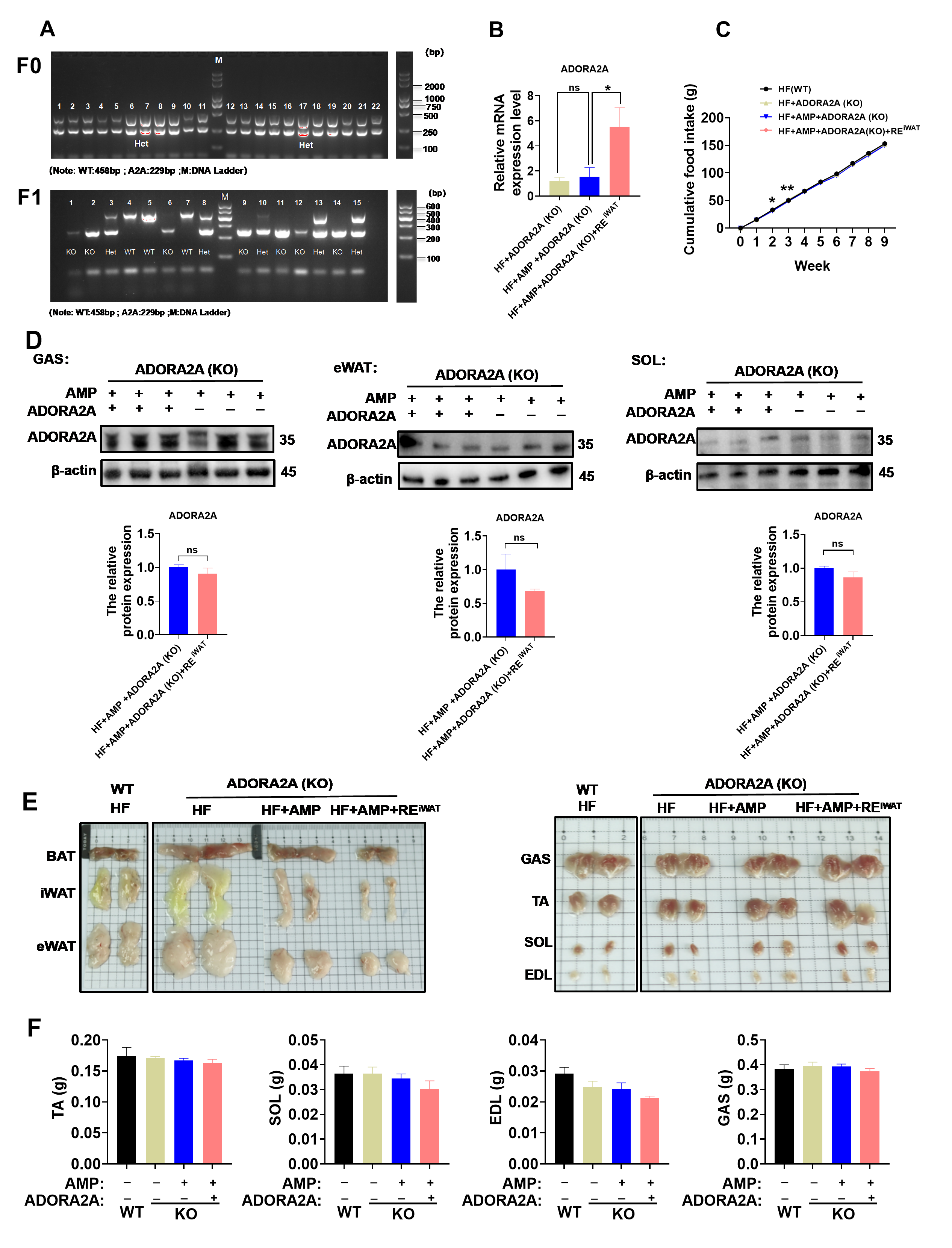


Figure S6. ADORA2A adenovirus supplementation can reproduce the role of AMP in lipid metabolism in ADORA2A knockout (KO) mice. (A) Genotypic identification of mice. (B) Real-time fluorescence quantification of ADORA2A in iWAT (*n* = 6). (C) The accumulated feed intake of mice within 0-9 weeks (*n* = 6-8). (D) Western blotting and quantification of ADORA2A in GAS, eWAT and SOL (*n* = 3), respectively. (E) The schematic map of fat and muscle of mice. (F) The weight of GAS, SOL, EDL, and TA in mice (*n* = 6-8), respectively. Data were analyzed by one-way ANOVA followed by post hoc Tukey’s tests and presented as mean ± SEM, ^ns^*p* > 0.05, In Figure S6C, ^*^ represents the comparison of HF+ADORA2A (KO) and HF+AMP+ADORA2A (KO), ^*^*p* < 0.05, and ^**^*p* < 0.01. AMP, adenosine monophosphate; BAT, brown fat; EDL, extensor digitorum longus; eWAT, epididymal white adipose tissue; GAS, gastrocnemius muscle; iWAT, inguinal white adipose tissue; SOL, soleus muscle; TA, tibialis anterior muscle.

Table S1 Diet composition of mice

| Items | CON | HF | HF+ADO | HF+AMP |
| --- | --- | --- | --- | --- |
| Composition (% (w/w)) |  |  |  |  |
| Casein | 19.0 | 25.8 | 25.8 | 25.8 |
| Corn Starch | 48 | - | - | - |
| Maltodextrin | 11.8 | 16.2 | 16.2 | 16.2 |
| Sucrose | 6.5 | 8.9 | 8.9 | 8.9 |
| Soybean Oil | 2.4 | 3.2 | 3.2 | 3.2 |
| Pork oil | 1.9 | 31.7 | 31.7 | 31.7 |
| Mixed minerals | 0.9 | 1.3 | 1.3 | 1.3 |
| CaHPO_4_ | 1.2 | 1.7 | 1.7 | 1.7 |
| CaCO_3_ | 0.5 | 0.7 | 0.7 | 0.7 |
| Potassium citrate | 1.6 | 2.1 | 2.1 | 2.1 |
| Mixed Vitamins | 0.9 | 1.3 | 1.3 | 1.3 |
| Cysteine | 0.3 | 0.4 | 0.4 | 0.4 |
| Choline bitartrate | 0.2 | 0.3 | 0.3 | 0.3 |
| ADO^b)^ | - | - | 0.1% | - |
| AMP^c)^ | - | - | - | 0.1% |
| Energy |  |  |  |  |
| AFE (kcal AFE/kg)^a)^ | 3845.5 | 5243.21 | 5243.21 | 5243.21 |
| AFE from fat (% kcal) | 10 | 60 | 60 | 60 |
| AFE from protein (% kcal) | 20 | 20 | 20 | 20 |
| AFE from carbohydrate (% kcal) | 70 | 20 | 20 | 20 |

^a)^(AFE, Atwater fuel energy); ^b)^(ADO, adenosine); ^c)^(AMP, adenosine monophosphate).

Table S2 The gene primer table of mouse

| Gene | Primer |
| --- | --- |
| β-actin | F: AGATGACCCAGATCATGTTTGAGA |
|  | R: CACAGCCTGGATGGCTACGT |
| ACC^a)^ | F: TTCAGTTCATGCTGCCCACA |
|  | R: AGGTTGGAGGCAAAGGACAT |
| ATGL^b)^ | F: GCCACTCACATCTACGGAGC |
|  | R: CCACGGATGGTGTTC |
| CD36^c)^ | F: TTTGACCCAGCACTTGAA |
|  | R: CAGTTAGGAGTCCGATGA |
| CREPα^d)^ | F: GGTTTAGGGATGTTTGGGTTTTT |
|  | R: AAGCCCACTTCATTTCATTGGT |
| FATP4^e)^ | F: ACTGTTCTCCAAGCTAGTGCT |
|  | R: GATGAAGACCCGGATGAAACG |
| HSL^f)^ | F: GCCGGTGACGCTGAAAGTGGT |
|  | R: CGCGCAGATGGGAGCAAGAGGT |
| PPARγ^g)^ | F: CCATTCTGGCCCACCAAC |
|  | R: AATGCGAGTGGTCTTCCATCA |

^a)^(ACC, acetyl-CoA carboxylase); ^b)^(ATGL, adipose triglyceride lipase); ^c)^(CD36, fatty acid translocase); ^d)^(CEBPα, CCAAT-enhancer-binding protein alpha); ^e)^(FATP4, fatty acid transporter protein 4); ^f)^(HSL, hormone-sensitive lipase); ^g)^(PPARγ, peroxisome proliferator-activated receptor γ).

Table S3 The antibody information table

| Gene |  |  |
| --- | --- | --- |
| β-actin | CST | 4970, dilution 1: 2000 |
| ADK^a)^ | Abcam | ab307357, dilution 1: 1000 |
| CD73^b)^ | Abcam | ab313339, dilution 1: 1000 |
| DNMT1^c)^ | Abcam | ab188453, dilution 1: 1000 |
| DNMT3A^d)^ | Abcam | ab188470, dilution 1: 1000 |
| DNMT3B^e)^ | Abcam | ab79822, dilution 1: 1000 |
| HSL^f)^ | Abcam | ab76492, dilution 1: 1000 |
| ADORA1 | Abcam | ab288377, dilution 1: 2000 |
| ADORA2A | Abcam | ab3461, dilution 1: 1000 |
| ADORA2B | Abcam | ab229671, dilution 1: 1000 |
| ADORA3 | Abcam | ab197350, dilution 1: 100 |

^a)^(ADK, adenosine kinase); ^b)^(CD73, ecto-5'-nucleotidase); ^c)^(DNMT1, DNA methyltransferase 1); ^d)^(DNMT3A, DNA methyltransferase 3A); ^e)^(DNMT3B, DNA methyltransferase 3B); ^f)^(HSL, hormone-sensitive lipase).

Table S4 The MSP methylation primer table

| Gene | Primer |
| --- | --- |
| MP^a)^-HSL^c)^ | F:GGAAAGGAGGTGATTAGTTTTAGTAC |
|  | R:AACAAAAATAACAAATACAACCGAC |
| UMP^b)^-HSL^c)^ | F:AGGAGGTGATTAGTTTTAGTATGG |
|  | R:AACAAAAATAACAAATACAACCAAC |

^a)^(MP, methylated primer); ^b)^(UMP, unmethylated primer); ^c)^(HSL, hormone-sensitive lipase).
